# Treatment discontinuation and re-initiation of anti-PD-(L)1 agents in metastatic cancers

**DOI:** 10.1007/s00432-020-03217-7

**Published:** 2020-04-18

**Authors:** Antti Tikkanen, Sanna Iivanainen, Jussi P. Koivunen

**Affiliations:** Department of Oncology and Radiotherapy, Oulu University Hospital and MRC Oulu, P.B. 22, 90029 Oulu, Finland

**Keywords:** Immune checkpoint inhibitor, PD-1, Re-challenge, Advanced cancer, Therapy discontinuation

## Abstract

**Introduction:**

Immune checkpoint inhibitors (ICIs) are approved in multiple indications for cancer care. Most of the clinical trials have not questioned shorter than until disease progression approaches. In this study, we present results from a cohort of multiple advanced cancers treated with restricted anti-PD-(L)1 therapy.

**Methods:**

All patients with advanced cancers treated with anti-PD-(L)1 therapy outside clinical trials at Oulu University Hospital 2014–19 were retrospectively identified from pharmacy records. Clinical variables, treatment history and survival were collected.

**Results:**

106 patients with median age of 66 years with lung cancer (*n* = 45, 42.5%), melanoma (*n* = 30, 28.3%), renal and bladder cancers (GU cancers) (*n* = 26, 24.5%), head and neck (H&N) cancer (*n* = 4, 3.8%), and colorectal cancer (*n* = 1, 0.9%) were included in the study. The median (m) OS for the whole population was 14 months (CI 9.7–18.3), 9 months (CI 6.3–11.7) for patients with no IO-free period (*n* = 64, 62.1%), and 27.0 months (CI 20.6–33.4, *p* = 0.000001) for patients (*n* = 39) with IO-free period. The mIO-free survival was 10.0 months (CI 7.1–12.9) for the whole cohort, 8.0 months (CI 1.7–14.3) for lung cancer, 23.0 months (CI 2.6–43.4) for melanoma, and 14.0 months (CI 0.0–20.4) for GU cancer. From the IO-free cohort, 19 patients needed re-treatment during follow-up, of which 8 were re-challenged with anti-PD-(L)1 therapy. The clinical benefit rate of anti-PD-(L)1 re-challenge was 37.5%.

**Conclusions:**

Our study shows that long IO-free periods can be achieved with limited duration of anti-PD-(L)1 therapy with excellent survival outcomes, and that anti-PD-(L)1 re-challenge is feasible in clinical practice.

## Introduction

Immune checkpoint inhibitors (ICIs) have been approved for treatment of advanced cancers as well as in adjuvant setting for stage III non-small cell lung cancer (NSCLC) after chemoradiation and high-risk resected melanoma (Antonia et al. 2017; Eggermont et al. 2018; Weber et al. 2017). There are currently several anti-PD-(L)1 therapy schemas in various advanced cancers (Balar et al. 2017; Borghaei et al. 2015; Brahmer et al. 2015; Gandhi et al. 2018; Hellmann et al. 2019; Herbst et al. 2016; Horn et al. 2018; Motzer et al. 2018,2015; Reck et al. 2016; Rini et al. 2019; Rittmeyer et al. 2017; Robert et al. 2015a, b) with similar treatment outcomes and toxicity profiles, yet, small subgroups of patients such as advanced NSCLC patients with PD-L1 expression ≥ 50% treated in first line, or tumors with MSI status seem to benefit more from specific therapies.

Due to the unique mechanism of anti-PD-(L)1 therapies, some patients experience long-lasting and durable responses, while a growing data shows that ICI re-challenge can bring meaningful clinical benefit to patients whose anti-PD-(L)1 therapy has been discontinued (Blasig et al. 2017; Fujita et al. 2020; Iivanainen and Koivunen 2019; Niki et al. 2018; Watanabe et al. 2019). Determination of the optimal treatment duration of ICIs has been studied rather minimally. Most of the clinical trials have investigated anti-PD-(L)1 agents until disease progression or severe side effects. One randomized study on advanced NSCLC patients has investigated discontinuing anti-PD-1 therapy at a 1-year fixed time. The results showed that while PFS was inferior for the patients in the discontinued treatment group, the OS was similar (Spigel et al. 2017). Our institutional guideline has restricted the maximal anti-PD-L(1) therapy duration to 6 months in advanced cancers, thus, providing a unique cohort to investigate anti-PD-L(1) therapy with restricted duration. We have previously reported from a small cohort of metastatic cancers that non-inferior outcomes can be achieved with early PD-1 agent discontinuation (Iivanainen and Koivunen 2019).

In this study, we investigate the treatment outcomes of restricted duration of anti-PD-(L)1 therapy with a larger cohort of patients. In addition, we define a novel outcome, immune-oncology (IO)-therapy-free survival (Iivanainen and Koivunen 2019) which captures the unique features of ICI responses, and gives a measure to assess the clinically meaningful benefit of anti-PD-(L)1 treatment.

## Materials and methods

### Patients

All the cancer patients who had received at least one dose of intravenous anti-PD-(L)1 inhibitor therapy at Oulu University Hospital in 2014–2019 were retrospectively identified from the pharmacy records. Patient’s age, date of diagnosis, date of metastatic disease, TNM staging, histology, molecular status of the tumor, adjuvant/metastatic treatment regimens, treatment-related adverse events, tumor responses, date of progression, and date of death/last follow-up were collected manually from the electronic patient records. Progression-free (PFS) and overall survival (OS) were calculated from the first date of anti-PD-(L)1 treatment to the documented tumor progression, death or end of follow-up (PFS) or to death or end of follow-up (OS). Tumor progression and/or death was counted as an event. Patients whose anti-PD-(L)1 treatment was discontinued in at least stable disease (SD) because of adverse events, complete response (CR), or maximal institutional recommended treatment length (6 months) were subjects to immuno-oncology (IO)-therapy-free survival analysis. IO-therapy-free survival was calculated from the last dose of anti-PD-1 therapy to the next treatment regimen, death or end of follow-up, of which the first two were counted as events. Tumor responses were retrospectively analyzed from electronic health records by two independent investigators (SI and JPK) with 100% concordance.

Data collection was carried out according to national legislation and under a permit from the medical director of Oulu University Hospital (study no. 299/2016). Anonymization was carried out before data analysis. Informed consent was not sought due to the register nature of the study.

### Statistical analysis

IBM SPSS Statistics 24.0.0.0 for Windows was applied for statistical analysis. Survival was analyzed by using the Kaplan–Meier method with the log-rank test. Probability values below 0.05 were considered significant.

## Results

### Demographics

Based on pharmacy records of Oulu University Hospital, a total of 106 patients with advanced stage cancer had received single-anti-PD-(L)1 therapies between August 2014 and August 2019 in outpatient settings and were included in the statistical analysis. The median age of the patients was 66 years and the majority of the patients (65.1%) were male. The cohort included patients with lung cancer (*n* = 45, 42.5%), melanoma (*n* = 30, 28.3%), renal and bladder cancers (genitourinary, GU cancer) (*n* = 26, 24.5%), head and neck (H&N) squamous cell carcinoma (*n* = 4, 3.8%), and one patient with colorectal cancer (0.9%). Most of the patients (91.5%) had stage IV disease and ECOG 0–1 (98.1%) performance status. Anti-PD-(L)1 therapy was given as a first-line therapy to a majority of the melanoma patients (73.3%), while 31 patients (68.9%) with lung cancer received the therapy in second line. All the other patients received the therapy in second or later line. The detailed demographics are presented in Table 1.

**Table 1:** Patient demographics and survival of the whole cohort

	*n* (%)
Age (median)	66
Gender	
Male	69 (65.1)
Female	37 (34.9)
Tumor type	
Lung cancer	45 (42.5)
Melanoma	30 (28.3)
GU cancer	26 (24.5)
H&N	4 (3.8)
CRC	1 (0.9)
Stage at diagnosis	
Stage IV	97 (91.5)
Other	9 (8.5)
ECOG	
0	48 (45.3)
1	56 (52.8)
2	1 (1.9)
Median OS (mo)	14.0
Lung cancer	13.0
Melanoma	21.0
GU cancer	14.0

### Overall survival of the whole cohort and patients with IO-free period

**Fig. 1:**
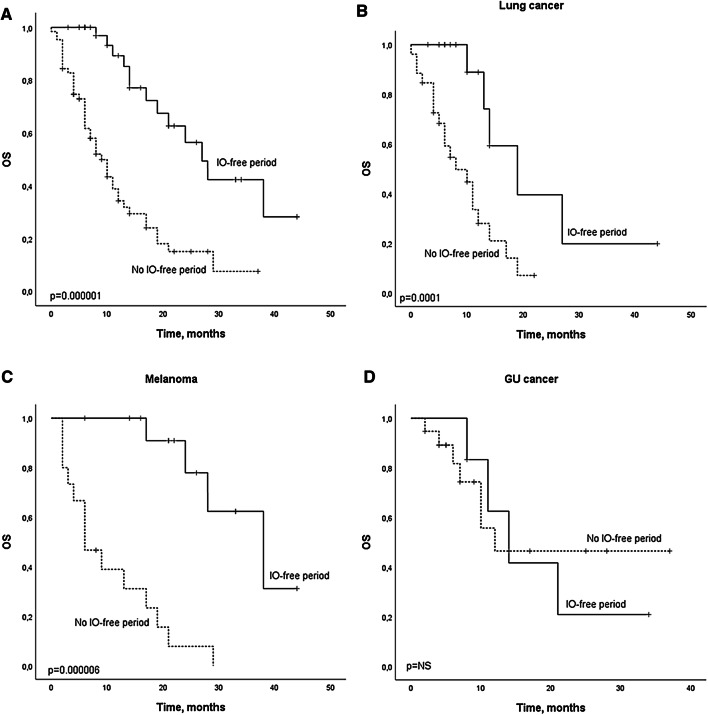
Kaplan–Meier analysis for the survival according to the presence (IO-free period) or absence (no IO-free period) of anti-PD-(L)1 therapy discontinuation in response in **a** the whole cohort **b** lung cancer, **c** melanoma, and **d** GU cancers. Crosses indicate censored events

From the 106 patients identified from the electronic healthcare records, 103 were eligible for survival analysis while three patients had non-assessable treatment response due to recent (< 3 moths) anti-PD-(L)1 therapy initiation. The median follow-up time was 8 months (CI 0–44.0). The overall survival was analyzed for the whole cohort (*n* = 103) and based on the status of the anti-PD-(L)1 therapy discontinuation. Patients whose anti-PD-(L)1 therapy was discontinued in at least SD response because of adverse events, CR, or maximal institutional recommended treatment length (six months), formed the IO-free cohort (*n* = 39). The median OS for the whole population was 14 months (CI 9.7–18.3) and for patients with no IO-free period (*n* = 64, 62.1%), the mOS was 9 months (CI 6.3–11.7), while for patients with IO-free period (37.9%) the median overall survival was 27.0 months (CI 20.6–33.4) (Fig. 1a, *p* = 0.000001). We also analyzed survival according to disease type based on the IO-therapy-free categorization. Among lung cancer patients, there were 19 patients (48.7%) with IO-free period, whereas 14 (35.9%) melanoma and 6 (15.4%) GU cancer patients had anti-PD-(L)1 therapy discontinued at least in SD at the six months time point. Median OS for the whole lung cancer cohort was 13.0 months and there was a statistical difference (*p* = 0.0001) between the 19 patients with IO-free period (19.0 months, CI 10.3–15.7) and patients with no IO-free period (8.0 months, CI 2.3–13.7) (Fig. 1b). Median OS for all the melanoma patients was 21.0 months (CI 11.7–30.3). For patients with IO-free period (*n* = 14), the median OS was 38 months (CI 23.0–53.0), while the overall survival of patients with no IO-free period was much lower, only six months (*p* = 0.000006) (Fig. 1c). Probably due to the rather low number of GU cancer patients with IO-free period (*n* = 6), the subgroup analysis was statistically non-significant (Fig. 1d). Patient demographics of the IO-free survival cohort are presented in Table 2.

**Table 2:** Patient demographics and survival of the IO-free cohort

	*n* (% or CI)
Age (median)	65
Gender	
Male	27 (69.2)
Female	12 (30.8)
Tumor type	
Lung cancer	19 (48.7)
Melanoma	14 (35.9)
GU cancer	6 (15.4)
Stage at diagnosis	
Stage IV	32 (82.1)
Other	7 (17.9)
ECOG	
0	23 (59.0)
1	16 (41.0)
Median duration of IO-treatment (mo)	3.0
Median IO-free survival (mo)	10.0 (7.1–12.9)
Lung cancer	8.0 (1.7–14.3)
Melanoma	23.0 (2.6–43.4)
GU cancer	10.0 (0.0–20.4)
Median OS (mo)	27.0 (20.6–33.4)
Lung cancer	19.0 (8.9–29.1)
Melanoma	38.0 (23.0–53.0)
GU cancer	14.0 (7.7–20.3)

### IO-therapy-free survival

**Fig. 2:**
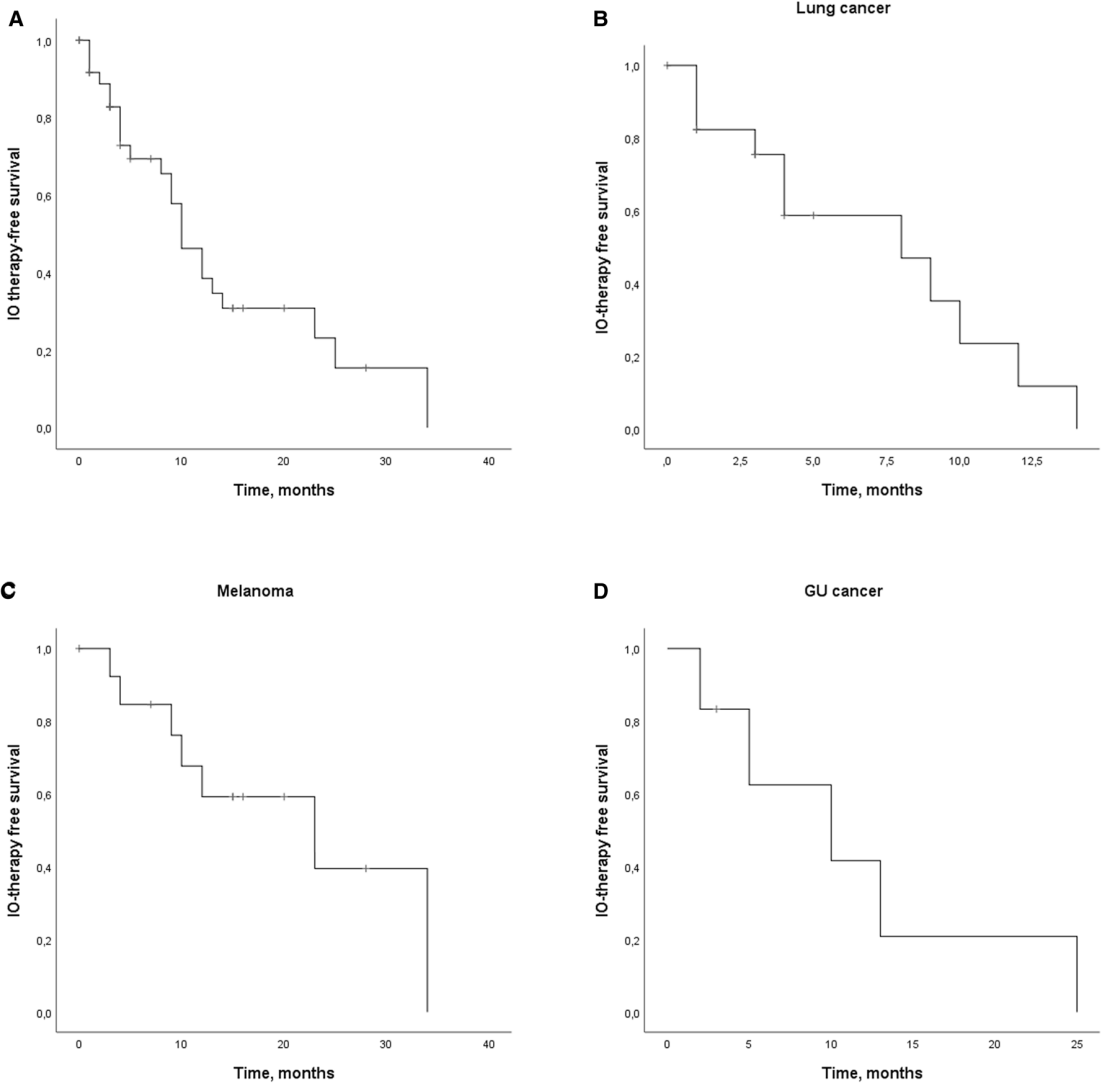
Kaplan–Meier analysis for the IO-therapy-free survival for **a** the whole cohort **b** lung cancer, **c**, melanoma and **d** GU cancer, whose anti-PD-(L)1 treatment was discontinued in response. Crosses indicate censored events

Patients who had at least SD response after six months of anti-PD-(L)1 therapy initiation were included in the IO-therapy-free survival analysis. The IO-free survival was defined as the length of the time from the last infusion of anti-PD-(L)1 therapy to the initiation of next treatment regimen, death or end of follow-up, the first two counted as events. The characteristics of the patients whose anti-PD-(L)1 therapy was discontinued in clinical response are presented in Table 3. Anti-PD-(L)1 therapy was discontinued in majority of the patients (71.8%) because of the maximal institutional-recommended treatment duration, whereas adverse events were counted for ~ 25% of the therapy discontinuations. Median duration of ICI therapy was 3.0 months and at the time of therapy discontinuation, five patients had CR (12.8%), 10 PR (25.6%), and 24 SD (61.6%) as disease status. With median follow-up time of 5 months (CI 0–34.0), the median IO-free survival was 10.0 months (CI 7.1–12.9) for the whole cohort, 8.0 months (CI 1.7–14.3) for lung cancer, 23.0 months (CI 2.6–43.4) for melanoma patients, and 14.0 months (CI 0.0–20.4) for GU cancer (Fig. 2a–d).

**Table 3:** Characteristics of patients whose anti-PD-(L)1 therapy was discontinued in response

	*n* (%)
Reason for IO discontinuation	
Adverse events	10 (25.6)
Complete response	1 (2.6)
Institutional recommended treatment duration	28 (71.8)
Disease status at discontinuation	
	CR 5 (12.8)
	PR 10 (25.6)
	SD 24 (61.6)
Treatment continuation after IO discontinuation	
No	16 (41.0)
Yes	19 (48.7)
Re-treatment modalities	
Anti-PD-1 therapy	8 (42.1)
Radiotherapy	7 (36.8)
Chemotherapy	3 (15.8)
TKI	1 (5.3)
Response rates after anti-PD-1 re-challenge	PR 1 (12.5)
	SD 2 (25.0)
	PD 5 (62.5)

### Re-treatment of the IO-free cohort

During the follow-up period, 16 patients (41.0%) from the IO-free cohort had no need for further therapy initiation. Re-treatment modalities for patients (*n* = 19, 48.7%) whose disease required re-treatment included anti-PD-(L)1 therapy re-challenge (*n* = 8, 42.1%), palliative radiotherapy (*n* = 7, 36.8%), chemotherapy (*n* = 3, 15.8%), and tyrosine kinase inhibitor therapy (*n* = 1, 5.3%). Four patients died without any further therapy. After the anti-PD-(L)1 re-challenge, the response rates included one PR (lung cancer) (12.5%), two SD (25.0%) (GU cancer, melanoma), and five PD (62.5%) (three melanoma patients and two lung cancer patients). There was no correlation between the initial response to anti-PD-(L)1 therapy and re-challenge response. The patients with clinical benefit on the re-challenge had PR (*n* = 2) or CR (*n* = 1) as initial response.

## Discussion

By far, ICI monotherapies have changed the treatment landscape of many advanced cancers with durable and even complete responses with acceptable toxicity profile. However, ICIs create a substantial economic challenge due to the undefined benefitting patient pool and treatment duration. The response rates to ICI monotherapies are generally low ~ 10–30% in undefined populations and there is a lack of clinically relevant predictive biomarkers to enrich the benefitting population. Furthermore, the optimal treatment duration in responding patients remains to be studied, since the registration trials have investigated the use of these agents until PD or up to 2 years.

In the current study, we present real-world treatment outcomes from a cohort of over 100 advanced cancer patients treated with restricted duration of anti-PD-(L)1 therapy. We have previously reported outcome results in the same setting with limited number of cases and a short follow-up time generating uncertainties in the data. Our previous results suggested that the limited treatment length of anti-PD-(L)1’s is associated with a low risk of rapid disease progression after therapy discontinuation, and with excellent survival outcomes of the approach. Currently, there is only a single study that has investigated anti-PD-1 therapy discontinuation in response in randomized fashion. The results of this study suggested that therapy discontinuation increases the risk for disease progression, but does not worsen the survival. Even though this study is generally thought to be negative for anti-PD-1 therapy discontinuation in response due to PFS difference, we feel that overall survival should be the primary end point of a discontinuation study in the context of metastatic cancer. There is a very limited number of retrospective studies on restricted anti-PD-(L)1 therapy, some of these, however, suggesting the feasibility of the approach (Jansen et al. 2019). Nevertheless, there are data from prospective trials indicating that patients can experience ongoing benefit after treatment discontinuation also in the absence of PD or treatment-related toxicities (Long et al. 2018; Robert et al. 2018; Topalian et al. 2014). There is a strong need for high-quality evidence to define early stopping rules; hence, two prospective trials, STOP-GAP (NCT02821013) and DANTE (ISRCTN15837212), are recruiting metastatic melanoma patients to evaluate the optimal treatment duration and the role of re-challenge of anti-PD-1 therapy.

The major finding of our study is that median IO-free survival of patients is 10.0 months (CI 7.1–12.9), which is very close to our previous report of 12.0 months (CI 3.5–20.5). This generates further cumulating evidence that restricted anti-PD-(L)1 duration does not lead to a rapid disease progression after therapy discontinuation. Since we had a larger cohort of patients with therapy discontinuation, we were able to study IO-free survival also in various cancers. The median IO-free survival ranged from 23.0 with melanoma patients to eight months in the lung cancer cohort, reflecting disease-specific nature to anti-PD-(L)1 response. This is in line with previous works showing that progression-free survival after IO therapy discontinuation is inferior in lung cancer compared to melanoma (Jansen et al. 2019; Spigel et al. 2017).

In our cohort, over 60% of patients had SD as the best response before treatment discontinuation. Many have suggested that treatment discontinuation is feasible in CR, but whether this can be generalized into patients with other types of responses, is unknown. Our study suggests that treatment discontinuation is a viable option also in PR and SD responses. The median duration of anti-PD-(L)1 therapy in our cohort was three months, which might reflect the rather high incidence of irAEs leading to preliminary treatment discontinuation. Yet, in a registration trial of pembrolizumab in advanced melanoma, the median duration of therapy was six months, while the median time to objective response was under three months (Robert et al. 2018). In this study, the majority of the patients with CR as their best confirmed overall response had PR or CR already at their first radiologic response assessment, implying that treatment benefit could be evaluated already in the early course of therapy.

Compared to our previous report, we now had more follow-up time and data available on the patients who had progressed within the IO-free period. 49% of the patients required re-treatment after IO-free period, and 42% of these were re-challenged with anti-PD-(L)1 therapy. There was a clinical benefit rate of 38% in patients with anti-PD-(L)1 re-challenge, suggesting that patients can respond to anti-PD-(L)1 re-treatment. Our results mirror other RWD results of anti-PD-(L)1 re-challenge where disease control rate has varied from ~ 20% to a bit over 40% with NSCLC patients (Fujita et al. 2020; Niki et al. 2018; Watanabe et al. 2019). A recent retrospective study on melanoma patients treated mainly outside clinical trials, whose anti-PD-1 treatment had been discontinued for any reason, reported a response rate of only 14.7% of single-anti-PD-1, and ~ 25% to combination-ICI therapy re-challenge, while there was no causality between the initial best overall response to re-treatment response (Betof Warner et al. 2020). Since improved survival is the primary end point of oncological care, strong conclusions cannot be made on the low response rates of therapy re-challenge, especially considering the unique nature of immunotherapy responses which generally do not follow traditional oncological response measures.

Analysis for the overall survival suggested that even though anti-PD-(L)1 therapy length was restricted, the overall survival was not sacrificed. In our study, the median OS for the melanoma cohort was 21.0 months, which is in line with other published RWD studies of advanced melanoma patients treated in first line (Grude et al. 2019). Furthermore, our NSCLC cohort consisted mostly of patients treated with anti-PD-1 therapy in the second line with the median OS of 13.0 months. This follows closely the survival results of nivolumab arm (median OS of 12.2) in the registration trial Checkmate-057 (Borghaei et al. 2015). Expectedly, the survival for the patients with IO-free period was significantly better than for those without it. However, it would demand a randomized prospective trial setting to be able to make strong conclusions on the restricted therapy length to survival.

Financial toxicity is a growing problem in the field of oncology, especially through the wide-scale use of anti-PD-(L)1 therapies. Our study shows that long IO-free periods can be achieved with limited duration of anti-PD-(L)1 therapy with excellent survival outcomes. This makes a strong statement that the anti-PD-(L)1 therapy length of registration trials should be questioned and investigated.

## Data Availability

The datasets generated and/or analyzed during the current study are not publicly available, but are available from the corresponding author on request.
